# How Technology Tools Impact Writing Performance, Lexical Complexity, and Perceived Self-Regulated Learning Strategies in EFL Academic Writing: A Comparative Study

**DOI:** 10.3389/fpsyg.2021.752793

**Published:** 2021-11-03

**Authors:** Yangxi Han, Shuo Zhao, Lee-Luan Ng

**Affiliations:** ^1^Faculty of Languages and Linguistics, University of Malaya, Kuala Lumpur, Malaysia; ^2^Faculty of Foreign Studies, North China University of Water Resources and Electric Power, Zhengzhou, China; ^3^School of International Studies, Communication University of China, Beijing, China

**Keywords:** academic writing performance, lexical complexity, psychological study preferences, self-regulated learning, study needs, technology-mediated SRL

## Abstract

Students experience different levels of autonomy based on the mediation of self-regulated learning (SRL), but little is known about the effects of different mediation technologies on students' perceived SRL strategies. This mixed explanatory study compared two technology mediation models, Icourse (a learning management system) and Icourse+Pigai (an automatic writing evaluation system), with a control group that did not use technology. A quasi-experimental design was used, which involved a pre and post-intervention academic writing test, an SRL questionnaire, and one-to-one semi-structured student interviews. The aim was to investigate 280 Chinese undergraduate English as a foreign language (EFL) students' academic writing performance, lexical complexity, and perceptions of self-regulated strategies in academic writing. One-way ANCOVA of writing performance, Kruskal-Wallis test of lexical complexity, ANOVA of the SRL questionnaire, and grounded thematic content analysis revealed that, first, both Icourse and Icourse+Pigai provided significant support for the development of SRL strategies vs. the control group, although there was no significant difference between the two groups. Second, Icourse+Pigai-supported SRL was more helpful for improving students' academic writing performance because it enabled increased writing practice and correction feedback. Third, Icourse+Pigai-supported SRL did not significantly improve students' lexical complexity. In conclusion, we argue that both learning management systems and automated writing evaluation (AWE) platforms may be used to assist students' SRL learning to enhance their writing performance. More effort should be directed toward developing technological tools that increase both lexical accuracy and lexical complexity. We conclude that the technical tools used in this study were positively connected to the use of SRL techniques. However, when creating technologically mediated SRL activities, students' psychological study preferences should be considered.

## Introduction

It has been well established that technology-mediated self-regulated learning (SRL) plays an increasingly prominent role in the language learning process (Zhu et al., 2016). Previous research has indicated that students experience different levels of autonomy based on the mediation they are provided for SRL (Bouwmeester et al., 2019; van Alten et al., 2020). However, not enough is known about the effects of different technologies on students perceived self-regulated learning strategies. Technology-mediated SRL enables students by providing more personalized pre-class preparation or classroom study, after-class practice, or discussion via online platforms and tools that support numerous resources and analyze individual learner data (Tan, 2019). As technological advances occur, instructors may need to adjust teaching strategies or modify their teaching practices within classrooms (Golonka et al., 2014). Learners, in turn, need to adapt to changes in their self-learning processes and practices necessitated by different types of technological tools (Cancino and Panes, 2021). Students may experience different cognitive loads depending on the study devices that they use to complete an assignment (Ko, 2017). For example, Ko (2017) indicated that learners' working memory load may be influenced by their physical learning environment, which includes different allocations of learning resources and technologies. Therefore, it is vital to understand the effects of different technologies on students' SRL processes and practices to better address students' learning needs.

According to a previous review (Broadbent and Poon, 2015), relatively insufficient attention has been paid to the effects of technology-supported SRL on academic achievement in English academic writing programs in blended learning settings in higher education. Academic writing, which predominately involves the development of a thesis, demands complex cognitive processes that requires the effective development of SRL strategies (Lam et al., 2018). Technology changes the EFL writing process from paper-based to online and subsequently influences the development of cognitive strategies in writing (Cancino and Panes, 2021). Thus, understanding technology mediation in SRL is crucial to better support students with effective SRL strategies. However, it is unclear whether technology use changes would ultimately change learning outcomes.

In Chinese higher education, poor academic English writing quality remains an issue among undergraduate students, despite their having received at least 10 years of English instruction since primary education. For instance, students are reported to compromise complexity for accuracy in their writing. They tend to overuse basic vocabulary, such as public verbs (e.g., say, stay, talk) and vague nouns (e.g., people, things, society) and avoid using advanced words or misuse advanced words in their academic writing (Hinkel, 2003; Zuo and Feng, 2015). Furthermore, the over-emphasis of accuracy in Chinese national academic English writing tests for non-English disciplines in higher education, such as College English Test Band 4 (CET-4) and Chinese English Test Band 6 (CET-6), reinforces such behavior (Zhang, 2019). However, linguistic complexity is an essential parameter by which to assess quality of English writing (Treffers-Daller et al., 2018; Xu et al., 2019). Among the various aspects of linguistic complexity, lexical complexity is crucial, as supported by research evidence from Csomay and Prades (2018), who found that higher quality essays among their participants were those that displayed a more comprehensive vocabulary range. However, whether technology-mediated SRL effectively enhances lexical complexity in students' academic writing has seldom been mentioned in previous research (Broadbent and Poon, 2015). Therefore, effort should be directed toward determining how technology-mediated SRL may help students to produce high-quality academic writing.

To address the issues mentioned above, this study compared the effects of Icourse and Icourse+Pigai-supported SRL on writing performance, lexical complexity, and perceived self-regulated learning strategies. The Small Private Open Course (SPOC) learning management platform enables enriched exposure to authentic materials and provides online quizzes and discussion boards to support various learning subjects (Qin, 2019). However, improvement in EFL learning to write requires enriched exposure to learning input and repeated writing practice with corrective feedback (Gilliland et al., 2018). Pigai provides automatic writing evaluation (AWE) with instant feedback and revision suggestions for learners, which may supplement individual learners' needs for synchronous feedback while simultaneously reducing teachers' workload. Combining Icourse and Pigai does not necessarily improve students' writing performance and writing quality or enhance SRL. Since the combination of technology use represents an extra burden and demands higher cognitive load of students, the blend of technology use may lead to a decline in students' satisfaction with learning (Xu et al., 2019). Thus, an investigation is required to determine the effects of different technology-mediated SRL on EFL learners' writing performance and quality.

## Literature Review

### Self-Regulated Learning

SRL refers to self-formed ideas, feelings, and actions that help individuals achieve their objectives (Zimmerman and Schunk, 2001; Seifert and Har-Paz, 2020). Technology-mediated SRL facilitates learners with flexible learning models and improves their language learning outcomes and motivation (An et al., 2020). Prior studies primarily focused on the effectiveness of technology-enhanced language learning within classroom instruction (An et al., 2020). There is a lack of empirical investigation of the effect of technology-mediated SRL on improving language skills. Of the limited number of previous studies that addressed technology-enhanced SRL, most reported positive relationships to language learning outcomes. For instance, Öztürk and Çakiroglu (2021) compared two groups of university students with and without SRL strategies in flipped learning. The findings indicated that SRL facilitated learning English speaking, reading, writing, and grammar. Similarly, students with SRL capabilities exhibited enhanced learning outcomes in blended learning settings (Zhu et al., 2016). In contrast, Sun and Wang (2020) found low-frequency use of SRL strategies among 319 sophomores Chinese EFL students in processes of learning writing, although SRL strategies significantly predicted writing proficiency. The students were reported to lack practice in writing during classroom sessions due to large classroom size and limited class time (Sun and Wang, 2020).

In terms of the instrument to measure SRL, the Motivated Strategies Learning Questionnaire (MSLQ) is frequently used (Pintrich et al., 1993). The MSLQ has been shown to be valid and reliable for use among undergraduate students. The original MSLQ assesses cognitive, meta-cognitive, and resource management strategies (Broadbent, 2017). Cognitive strategies involve the preparation, elaboration, and management of studies. Meta-cognitive strategies primarily refer to self-control. Resource management includes time management, effort regulation, and peer learning (Broadbent, 2017). According to Broadbent's (2017) review of 12 SRL regulated online studies, meta-cognition and resource management strategies positively influence learning outcomes, while cognitive strategies have the least amount of empirical evidence to suggest their utility. As SRL theory developed from a focus on meta-cognition to recognizing its multifaceted nature, it included motivation factors that influence learning (An et al., 2020). Pintrich (2004) noted an issue of the MSLQ is that it does not include motivational and affective factors that determine essential emotional strategies (Pintrich, 2004; An et al., 2020). Therefore, this study adopted a revised version of the MSLQ that includes four SRL aspects: cognitive, metacognitive, resource management, and emotional strategies (Supplementary Appendix 1).

### Lexical Complexity

The ultimate goal for the technology-supported SRL, in this context, is to improve students' writing performance and writing quality. More specfically, lexical complexity is an essential indicator of EFL writing (Lemmouh, 2008; Zhu and Wang, 2013), but few studies have addressed the effect of SRL on linguistic complexity in EFL programs. O'Dell et al. (2000) suggested that lexical complexity primarily involves lexical diversity, lexical sophistication, and lexical density (the ratio of content words to tokens). The lexical diversity aims to measure lexical variability, while lexical sophistication compares the ratio of advanced words to the total tokens. Treffers-Daller et al. (2018) highlighted the importance of integrating lexical diversity, the range of words used to measure lexical complexity, and lexical sophistication, with reference to less frequently used words as defined by various standards. Previous literature on lexical complexity development is inconclusive; some studies discovered growth after training, while others did not (Knoch et al., 2014; Kalantari and Gholami, 2017). Bulté and Housen (2014) affirmed the possibility of capturing changes in linguistic complexity in L2 writing over a short period. Inquiry into the effects of various technologies on lexical complexity is necessary so that language teachers can support students with desirable technology-mediated SRL strategies, thus enabling students to achieve enhanced learning outcomes, such as better writing quality.

### Icourse and Pigai

**Figure 1:**
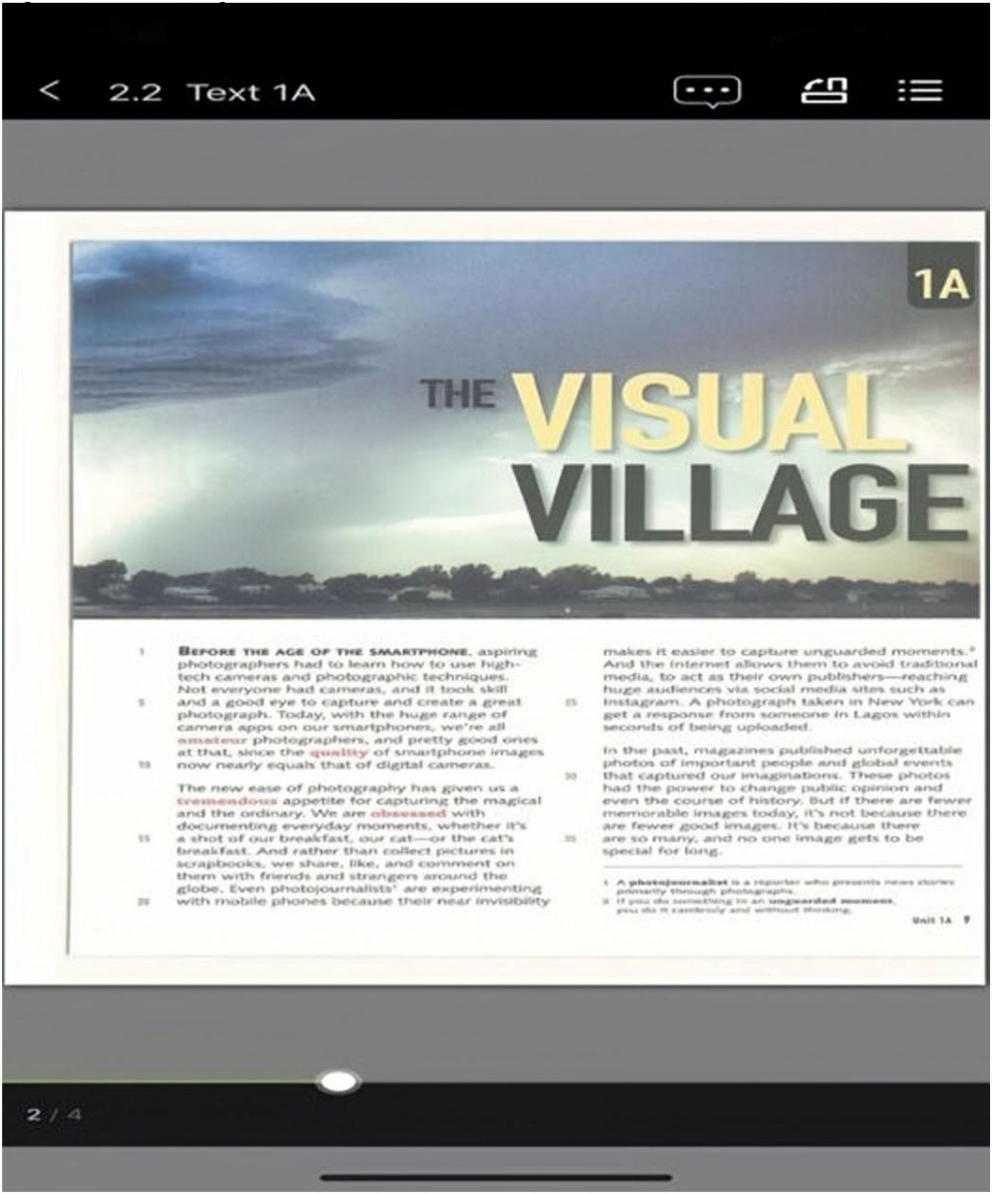
Icourse—Text preview screenshot.

**Figure 2:**
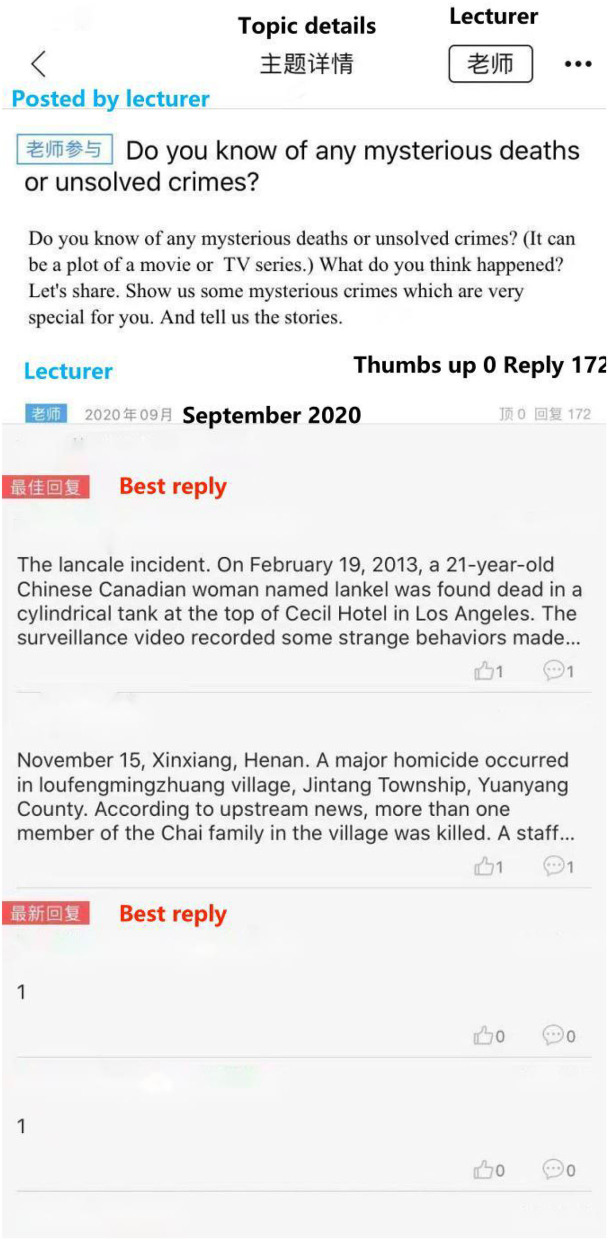
Icourse—Online discussion screenshot.

**Figure 3:**
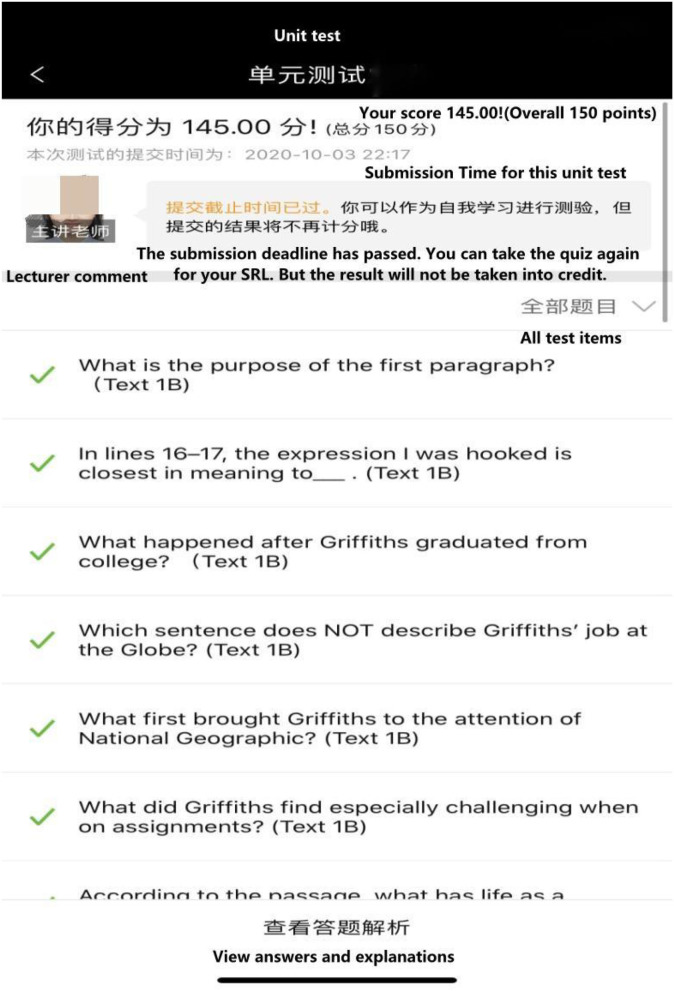
Icourse—Online quiz screenshot.

The technology tools adopted in the technlogy-mediated SRL in this research are the Icourse and Pigai. Based on previous studies (Golonka et al., 2014; Yang and Dai, 2015; Zhai, 2017), the definitions of Icourse and Pigai are presented in Table 1. Massive open online courses (MOOCs) are often criticized for high dropout rates and low student engagement (Gilliland et al., 2018; de Moura et al., 2021). Icourse, as a SPOC platform, is claimed to be a valid alternative as course designers, usually course lecturers, permit the course syllabus to be flexible in difficulty and more adaptable to different student characteristics (Ruiz-Palmero et al., 2020). Guo et al. (2021) quantitatively assessed the impact of the SPOC-based blended learning model embedded in the undergraduate course of International English Language Testing System (IELTS) writing at a Chinese university in Beijing. IELTS is an international standardized proficiency English test for non-English speakers. Assessments were made of writing performance through classroom observation, questionnaires, and achievement tests in pre, mid, and final terms. The experimental group outperformed the control group in the final term test results, but there were no significant differences in pre and mid-term results. However, the study did not include linguistic parameters for evaluating SPOC platforms' effects on EFL learning of writing. Of the few studies that did include linguistic measurement, Cheng et al. (2017) addressed the impact of SPOC learning management systems on 35 Chinese undergraduate EFL learners' writing performance in terms of essay length, accuracy, and lexical complexity. The findings revealed that the SPOC learning platform helped the learners to write with increased accuracy and fluency and with an increased ratio of advanced academic vocabulary in the post-test compared to the pre-test. However, the study did not include comparison with a control group that did not use the SPOC platform. Overall, prior studies highlighted the positive role SPOC platforms play in assisting the EFL learning of writing, in terms of improving writing test scores, accuracy, and fluency. Figures 1–3 illustrate how Icourse functions as a SPOC learning management system to support browsing course materials, answering online quizzes, and interacting via discussion boards.

**Table 1:** Definition of Icourse and Pigai.

1.	Icourse	Under the Small Private Online Course (SPOC) platform, course organizers use the platform to publish course content, learning activities and discussion topics; learners use various social learning tools, including course discussion spaces, course resource sharing tools, and online quizzes to participate in learning	Enable sharing of course materials that cater to students characteristics, allowing accessibility to content anytime, anywhere
			**Enable sharing of authentic MOOC videos, with higher quality and multiple choices**
			**Facilitate course content organization and teacher-student and student-student communication with online discussion boards**
			**Foster practice with online quizzes**
**2**.	**Pigai**	**Based on natural language processing technology and corpus technology, which analyzes the distance between students' compositions and the standard corpus to score students' English written essays instantly. Provides suggestions for improvement and content analysis**	**Provide immediate and large-scale online automatic corrections**
			Create student corpora based on the composition assignments submitted by students, and compare errors, word frequencies, collocations, graded vocabulary, data comparison, and dimensional analysis Support teacher manual correction function

**Figure 4:**
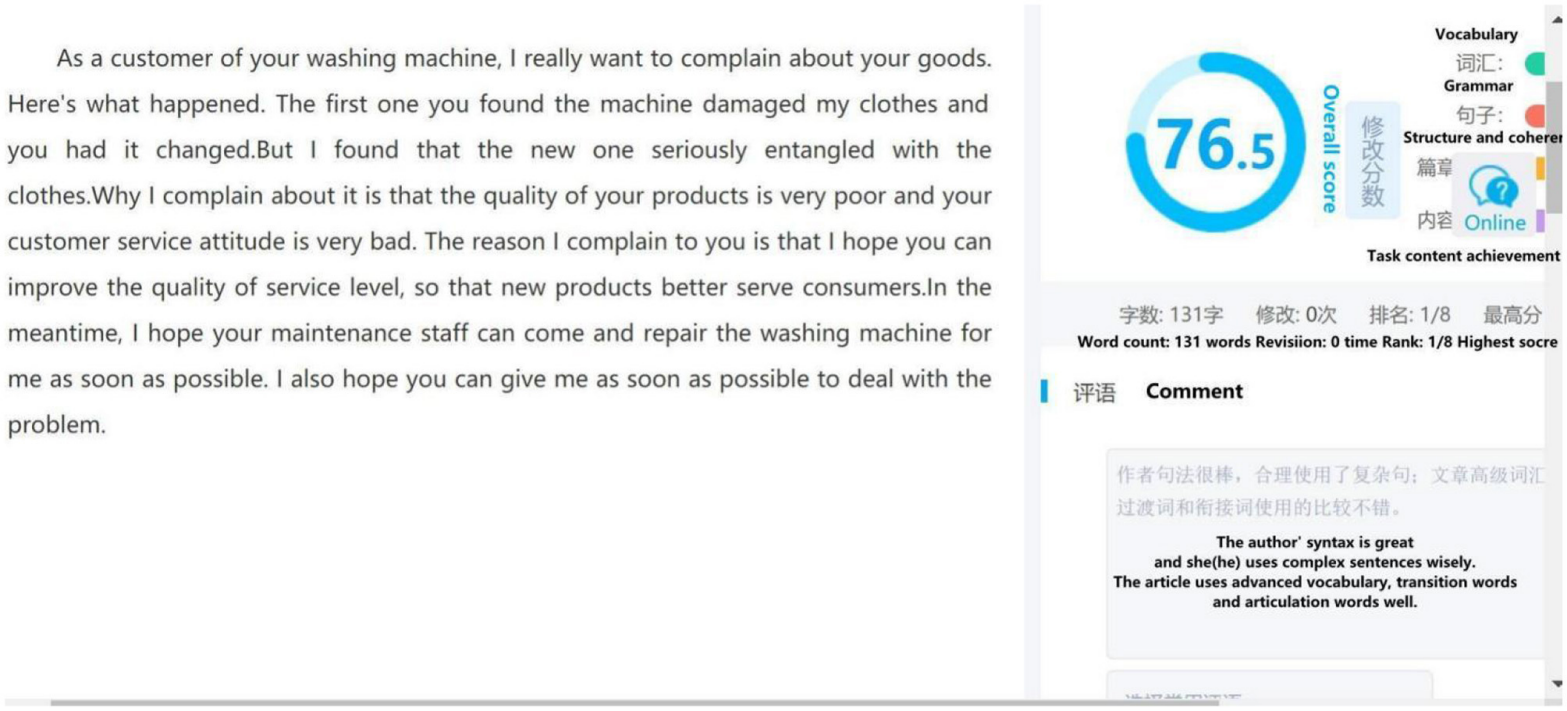
Pigai—Automatic writing evaluation screenshot.

**Figure 5:**
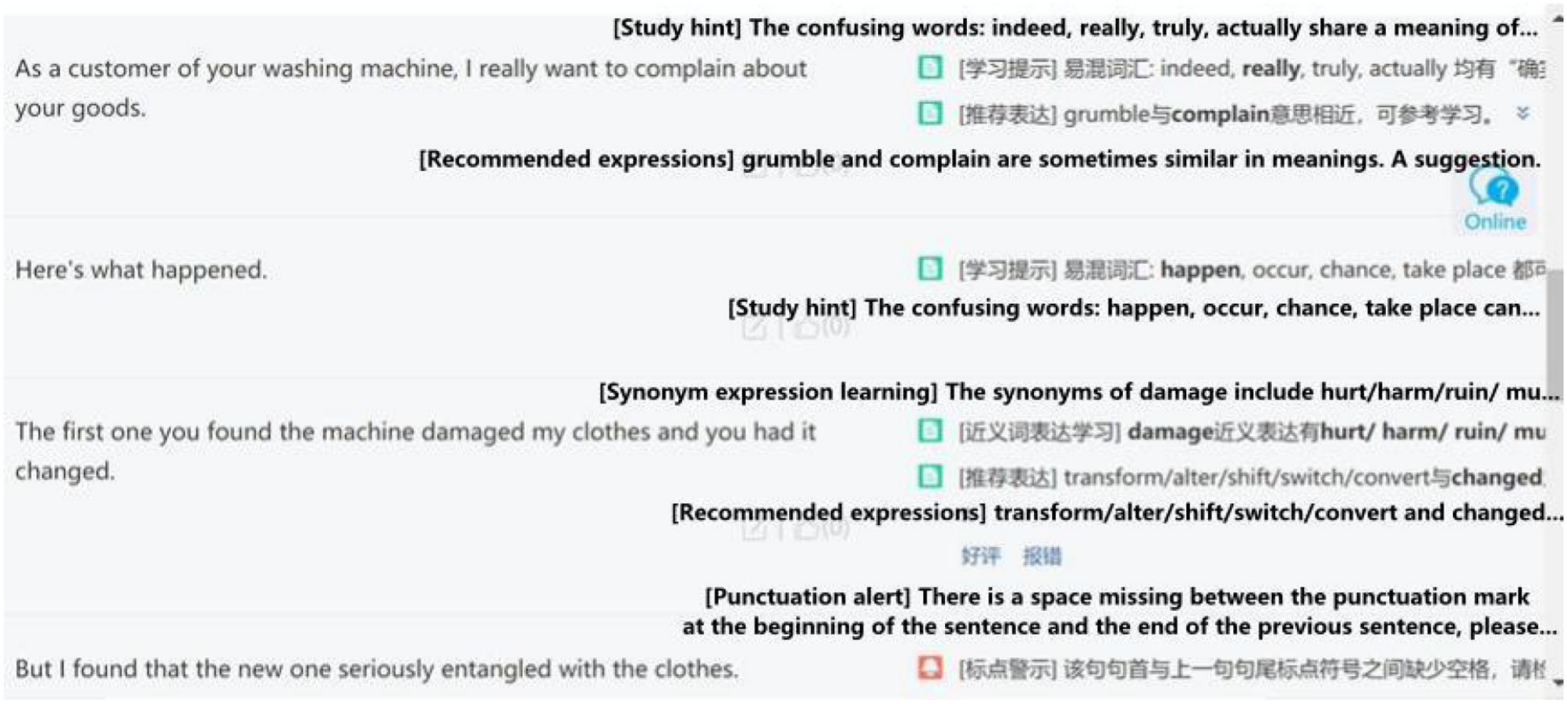
Pigai—Revision suggestions.

Besides the learning management system, Pigai is used as an AWE tool in this research. AWE aims to provide prompt writing revision feedback to learners (Liao, 2016). The major difference between AWE and teacher feedback is that AWE calculates the language gap between the EFL learner's language use and that of the native speaker (Li et al., 2019). While teacher feedback mainly relies on teachers' knowledge and teaching experience. Figures 4, 5 illustrate how Pigai works as an AWE tool to support learning how to write proficiently in English. Figure 4 shows how Pigai gives an overall mark to students' essays based on lexical, syntactic, semantic, and content parameters. A general remark on the vocabulary and sentence use is also displayed at the lower right corner of the screen. Figure 5 illustrates how Pigai provides detailed feedback regarding confusing words, synonyms, and convertible sentence patterns to expand students' vocabulary and sentence use.

Overall, positive findings support the applicability and efficiency of Pigai (Lin et al., 2020). For instance, Li and Zhang (2020) reported a positive role of Pigai in improving Chinese EFL learners' writing performance and writing self-efficacy by indicating errors in students' writing in real time and thereby enabling them to acquire vocabulary and sentence-construction knowledge. In contrast, some researchers have argued Pigai has deficits (Wu, 2017). For example, Pigai is less effective at providing feedback that helps logical thinking and content structure organization, which are also crucial factors for successful compositions, in addition to vocabulary and grammar (Wu, 2017). While the technology of Pigai constantly updates and adapts to emerging pedagogical needs, Hou (2020) called for additional studies of Pigai to keep pace with its technological advances.

Determining effective ways to support EFL learning is complex. Careful consideration should be made of the combination of various technologies, rather than favoring one specific technology over another (Lam et al., 2018).

According to the research aim, the research questions of the current study were as follows:

1. To what extent does technology-mediated SRL impact undergraduate Chinese EFL learners' written performance?
2. To what extent does technology-mediated SRL impact undergraduate Chinese EFL learners' written performance in terms of lexical complexity?
3. To what extent do technology tools impact undergraduate Chinese EFL learners' use of SRL strategies?
4. What factors impact undergraduate Chinese EFL learners' perceptions of using technology-mediated SRL during their writing in English?

## Materials and Methods

### Participants

Purposive sampling was used to recruit the participants. The initial plan was to recruit 300 sophomore students from water conservancy engineering, mechanical engineering, electronic engineering, and allied subjects. However, although the intention was to have 100 students in each group, only 280 students agreed to participate in this study. Of these students, 99 were assigned to the control group, 90 to the Icourse group, and 91 to the Icourse+Pigai group. The participants were from the same Henan province in the People's Republic of China to ensure that they shared a similar EFL learning background. Their average age was 19 years (SD = 1.169), and each had at least 10 years of EFL learning experience since their primary education.

The research complied with all ethical stipulations of the ethics committee at the University of Malaya. Before conducting the research, the relevant university administrators were fully informed, and all the students voluntarily participated in the study, and each signed an online consent form before participating in the study. All participants remained anonymous during the entire research process.

### Research Design

**Figure 6:**
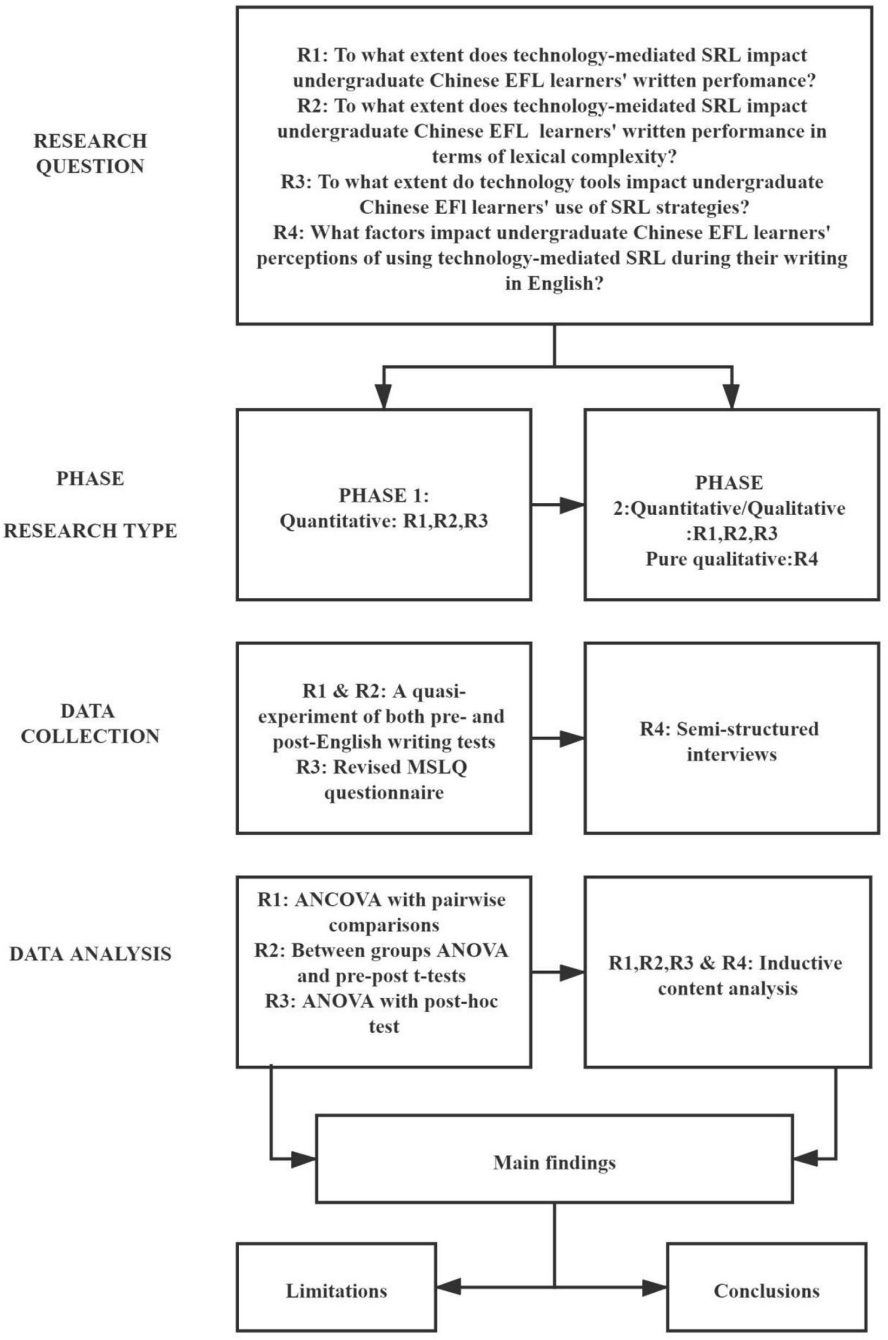
Flowchart-research process.

An explanatory sequential mixed-methods approach was used to address the research questions (Figure 6). First, a quasi-experimental study was conducted to obtain quantitative comparative data, with follow-up qualitative data derived from student interviews. This study assessed three groups: a control group that received no technology-mediated SRL, an Icourse-assisted SRL group, and an Icourse and Pigai supported SRL group. Icourse and Pigai supported self-regulated learning of academic writing both in and outside the classroom. The academic writing course outline is presented in Table 2. These systems include pre-class online learning of vocabulary, watching online instruction videos about writing skills, lead-in quizzes, sentence paraphrase practice, online forum discussion, after-class review, essay writing online submission, and receiving feedback and revisions. The same academic writing course was delivered to all three groups, using the same textbook in classroom instruction. The same five units of content were covered in one academic term and the class frequency was the same, namely three times per week for 90 mins per class. Each group was recruited from a general polytechnic-focused university. All three universities were ranked at the same level. The three senior lecturers in the three groups shared a similar educational background, namely, holding a master's degree and having taught 10–12 courses. Lecturers similarly monitored students' SRL processes by setting up tasks, quizzes, and activities with deadlines, and answered students' questions online when needed.

**Table 2:** The EFL academic writing course outline.

**Course: EFL academic writing course for second year non-English major students**			
**Course guideline: Preview+Lecture+Assignment+Assessment**			
**Course frequency: 4 classes per week (45 mins/per class)**			
	**Control**	**Icourse**	**Icourse+Pigai**
Preview (before classes)	With textbook	Icourse	Icourse+Pigai
Lecture (in-classes)	2 lectures+ 2SRL with textbook	2 lectures+2 SRL with Icourse	2 lectures+ 2SRL with Icourse+Pigai
Assignment (after classes)	In paper	Icourse	Icourse+Pigai
Assessment (both in and out of classes)	In paper	Icourse+paper	Icourse+Pigai+paper

#### Writing Performance

##### Instrument

The two composition topics were revised from the academic International English Language Testing System (IELTS) Writing Task 2 (Esol, 2007). The topic was “What is your opinion on consumer complaints?” The second was “What is your opinion on distance education?” The reasons for selection of these tasks were that, first, the IELTS Writing Task 2 focuses on academic writing. Second, the grading rubrics for IELTS cater more to the research purpose of measuring students combined lexical accuracy and complexity rather than simply focusing on lexical accuracy alone. Supplementary Appendix 2 presents the revised writing grading rubrics (Esol, 2007). A pilot study was conducted among 30 students to check validity and reliability. KMO was 0.6 and Bartlett's test *p* value were 0.00 and Cronbach's alpha was 0.79; these values were considered acceptable for the study.

##### Data Collection

Both pre and post-tests were delivered online through scanning Quick Response (QR) codes. Before delivering the tests, the participants signed an informed consent electronically by scanning QR codes. The pre-test was delivered at the beginning of the academic term in September 2020, and the post-test was given at the end of the academic term in January 2021. Anti-cheating measures and a time limit of 30 mins were enforced to avoid plagiarism. If the online submission was blank or highly suspected of plagiarism, this composition was considered invalid. The number of valid cases for each group was 73 (out of 99), 70 (90), and 72 (91).

##### Data Analysis

Both AWE graded the tests in Pigai, as well as the researcher, and another experienced teacher. The average grade of the three grading results was regarded as the final result for each participant. After grading, the pre-test results were used as covariates in a one-way ANCOVA of academic achievement. The effect size was calculated.

#### Lexical Complexity

##### Instrument

The student texts in each group were assessed in terms of their lexical diversity, lexical density, and lexical sophistication. The lexical diversity measure used the STTR (standard type-token ratio) as measured by Wordsmith 8.0, developed by Mike Scott. The WordSmith software was originally developed by University of Liverpool, UK, and published by Oxford University Press. The measurement of STTR is more accurate than the type-token ratio (TTR) because it is less dependent on the text length (Treffers-Daller et al., 2018). The lexical density and lexical sophistication were calculated using Range 32, designed by P. Nation and A. Coxhead. By calculating the ratios of content words to the total tokens, Range 32 first excludes the built-in function words (function text) as filter words and obtains lexical density ratios (Zhu and Wang, 2013). Range 32 uses Laufer and Nation's base word list for the most high-frequency words, the second 1,000-word list (hereinafter referred to as baseword 2) for the next most high-frequency words, and the third word list (hereinafter referred to as baseword 3) for advanced academic vocabulary (Laufer and Nation, 1995; Zhu and Wang, 2013). Lexical sophistication was measured by calculating the frequency of words other than Range 32, which refers to the ratio of defined baseline 2 and 3 words with no spelling errors to the total token (Gong et al., 2019).

##### Data Collection and Analysis

The texts were first processed to identify spelling errors and homographs. Misused words were removed from the essay entry process to ensure that all words entered were correct output words. Where words were selected correctly but spelt incorrectly or homographs, such as bat the animal or bat for baseball, the researchers corrected them and added a marker afterwards. A pre-test was conducted to examine whether there were any differences among the three groups in terms of lexical complexity before the intervention. Both between-groups and timewise comparisons were conducted to determine whether there was any significant effect of technology-supported SRL on lexical complexity. Since the Levene test hypothesis was violated, the post-test lexical complexity ratios were analyzed using SPSS 26 with Kruskal-Wallis tests.

#### SRL Strategies

##### Instrument

Based on the literature review, a revised version of the MSLQ was applied to measure the technology-mediated SRL strategies in this study (Supplementary Appendix 1). MSLQ was initially developed by the National Center for Research USA after completing numerous correlational research on SRL and motivation (Pintrich, 2003). The tool consists of four sections: emotional (including motivational and affective factors), cognitive (including elaboration, rehearsal, and organization), metacognitive (including self-control), and resource management (including time management and peer learning). A five-point Likert-type scale was adopted for the self-report questionnaire, with responses ranging from 1 (strongly disagree) to 5 (strongly agree). The questionnaire was in Mandarin to ensure that the participants could fully understand all items. A pilot test was conducted with 30 undergraduate students other than the participants, which revealed a Cronbach's alpha value of 0.899 and a value of 0.917 for KMO and Bartlett's test.

##### Data Collection and Analysis

Participants received access to the questionnaire through QR code scan. The questionnaire was collected only after the intervention. One-way ANOVA was conducted to determine whether there were any statistically significant differences among the three groups.

#### Technology Use Factors Toward Technology-Supported SRL

##### Instrument

One-to-one interviews (Supplementary Appendix 3) were conducted with 10 participants (Icourse: 5, Icourse+Pigai: 5) to explore the reasons for the quantitative data using in-depth evidence. The interviews were semi-structured, and follow-up questions such as “how” or “why” were added based on the interviewees' answers. The interviews were designed and delivered in Mandarin Chinese to ensure that the interviewees could understand all interview questions.

##### Data Collection and Analysis

Interviewees were randomly chosen from the experimental groups. Each interview required up to 30 mins through WeChat video chat. WeChat is a free application that Tencent launched on January 21, 2011 to provide instant messaging services. All interviews were recorded after obtaining the interviewees' permission. All records were transcribed verbatim and translated into English by a licensed professional translator. The transcripts were then coded and analyzed using Nvivo 12. Inductive content analysis was used because no predetermined codes were used. Based on preliminary analyses, the researcher established the relationships between the nodes and checked them against the data.

## Results

### Writing Performance

**Figure 7:**
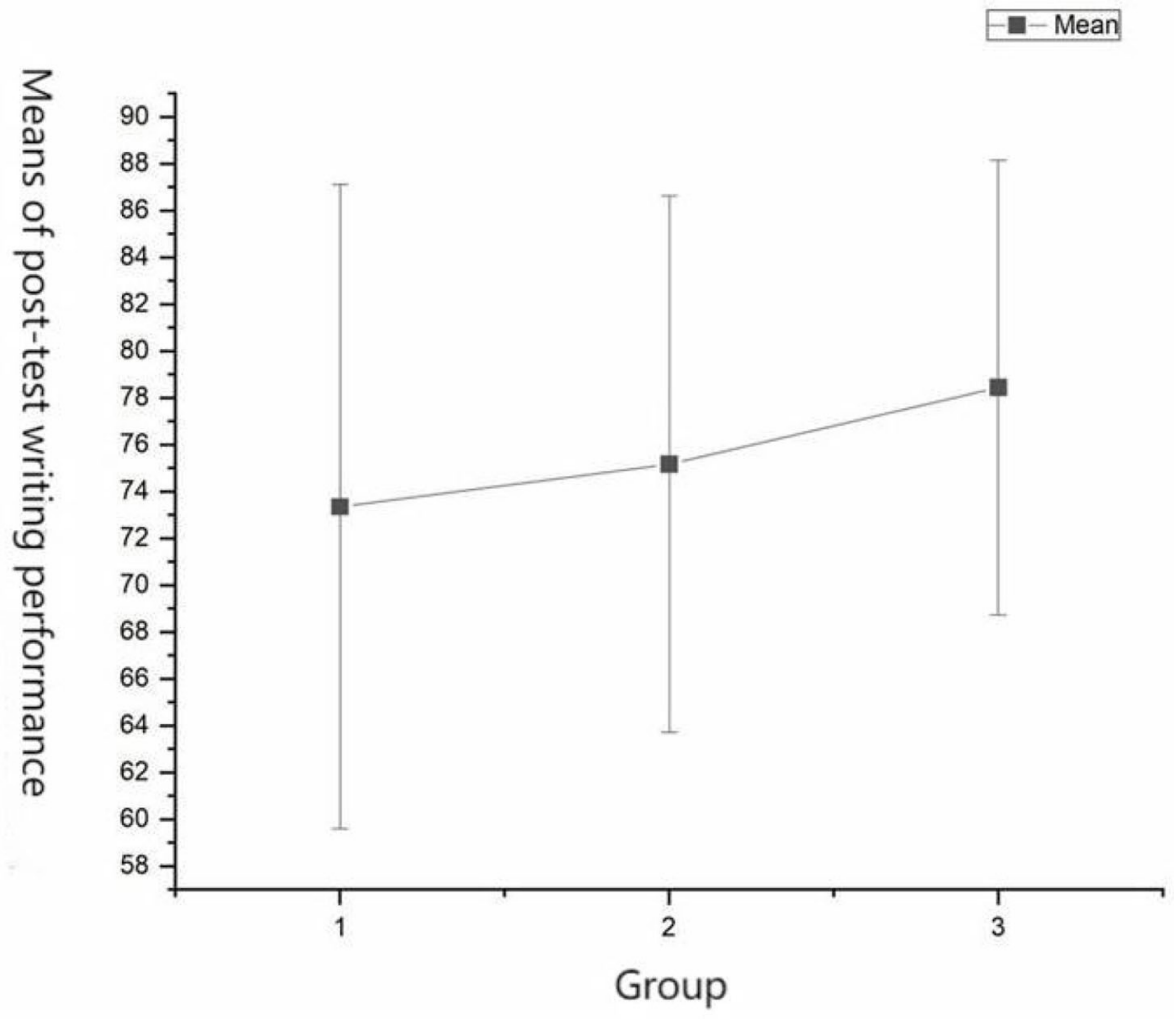
Means plot of post-test writing performance.

A one-way between-groups ANCOVA was conducted to compare the effectiveness of Icourse (group 2) and Icourse+Pigai (group 3) supported self-regulated learning on the participants' writing performance as compared to the control group (group 1) after one academic term. The independent variable was the technology tools used, and the dependent variable was the post-test score. Participants' pre-test scores were used as covariates in this analysis. Preliminary checks were conducted to ensure that the assumptions of normality, linearity, homogeneity of variances, homogeneity of regression slopes, and reliable measurement of the covariate were met. After adjusting for pre-test scores, there were significant differences in mean scores among the three groups (Figure 7) [*F*_(1, 211)_ = 4.03, df = 2, *p* = 0.019, partial η^2^ = 0.04; Table 3]. According to the pairwise comparisons shown in Table 4, the Icourse+Pigai group (*M* = 78.44, SD = 9.71) significantly outperformed the control group (*M* = 73.35, SD = 13.76; *p* = 0.01). The difference between the Icourse group (*M* = 75.16, SD = 11.46) and the control group (*M* = 73.35, SD = 13.76; *p* = 0.23) was not statistically significant.

**Table 3:** ANCOVA tests of between-subjects effects at post-test for writing performance.

**Source**	**Type III Sum of squares**	**Df**	**Mean square**	***F***	***p***	**Partial eta squared**
Corrected model	9,154.694^a^	3	3,051.565	30.361	<0.001	0.302
Intercept	20,936.195	1	20,936.195	208.302	<0.001	0.497
Pretest	8,192.335	1	8,192.335	81.509	<0.001	0.279
Group	809.980	2	404.990	4.029	0.019	0.037
Error	21,207.337	211	100.509			
Total	1,260,601.250	215				
Corrected total	30,362.030	214				

a *R^2^ = 0.302 (Adjusted R^2^ = 0.292)*.

**Table 4:** Pairwise comparisons at post-test writing performance.

**(I) group**	**(J) group**	**Mean difference (I-J)**	**Std. error**	***p* ^**a**^**	**95% confidence interval of difference**	
					**Lower bound**	**Upper bound**
1	2	−2.012	1.677	0.232	−5.318	1.294
	3	−4.714^*^	1.666	0.005	−7.997	−1.430
2	1	2.012	1.677	0.232	−1.294	5.318
	3	−2.702	1.684	0.110	−6.021	0.618
3	1	4.714^*^	1.666	0.005	1.430	7.997
	2	2.702	1.684	0.110	−0.618	6.021

* *The mean difference is significant at the 0.05 level*.

a *Adjustment for multiple comparisons: Least Significant Difference (equivalent to no adjustments)*.

### Lexical Complexity

Table 5 illustrates that the pre-test comparison indicated no statistically significant differences in lexical diversity, lexical density, or lexical sophistication among the three groups. After the intervention, significant differences were observed in all three lexical complexity indicators in the Icourse group, but only in lexical diversity and density in the Icourse+Pigai group after the intervention. The latter group showed no significant differences in lexical sophistication after the intervention. In contrast, the control group exhibited no statistically significant difference from pre-test to post-test for any of the three lexical complexity indicators.

**Table 5:** Between groups and pre- and post-test comparisons for lexical complexity.

**Lexical complexity**		**Diversity**			**Density**			**Sophistication**		
Between groups (Pre-test: one way ANOVA, posttest: Kruskal Wallis test)	*p* < 0.05	Control vs. Icourse	Control vs. Icourse+Pigai	Icourse vs. Icourse+Pigai	Control vs. Icourse+Pigai	Control vs. Icourse+Pigai	Icourse vs. Icourse+Pigai	Control vs. Icourse	Control vs. Icourse+Pigai	Icourse vs. Icourse+Pigai
	Pre-test	0.204	0.338	0.027	0.286	0.968	0.270	0.294	0.368	0.876
	Post-test	/	/	/	0.623	0.069	0.020	/	/	/
Pretest and posttest (independent t-test)	*p* < 0.05	Pretest vs. Posttest			Pretest vs. Posttest			Pretest vs. Posttest		
	Control	0.881			0.874			0.254		
	Icourse	0.016			0.003			0.000		
	Icourse+Pigai	0.002			0.013			0.102		

The Kruskal-Wallis post-test revealed no statistically significant differences in lexical diversity and sophistication across the three groups (control group, *n* = 73; Icourse group, *n* = 70; Icourse+Pigai group, *n* = 72), $\chi_{\left(2,215\right)}^{2}$ = 5.53, *p* = 0.063 (diversity), $\chi_{\left(2,215\right)}^{2}$ = 6.02, *p* = 0.049 (density), $\chi_{\left(2,215\right)}^{2}$ = 0.06, *p* = 0.970 (sophistication). The result leads the null hypothesis to be rejected that the distribution of lexical density is the same across the three groups, as there was a significant difference between the Icourse group and the Icourse+Pigai group in lexical density after the intervention.

### SRL Strategies

**Figure 8:**
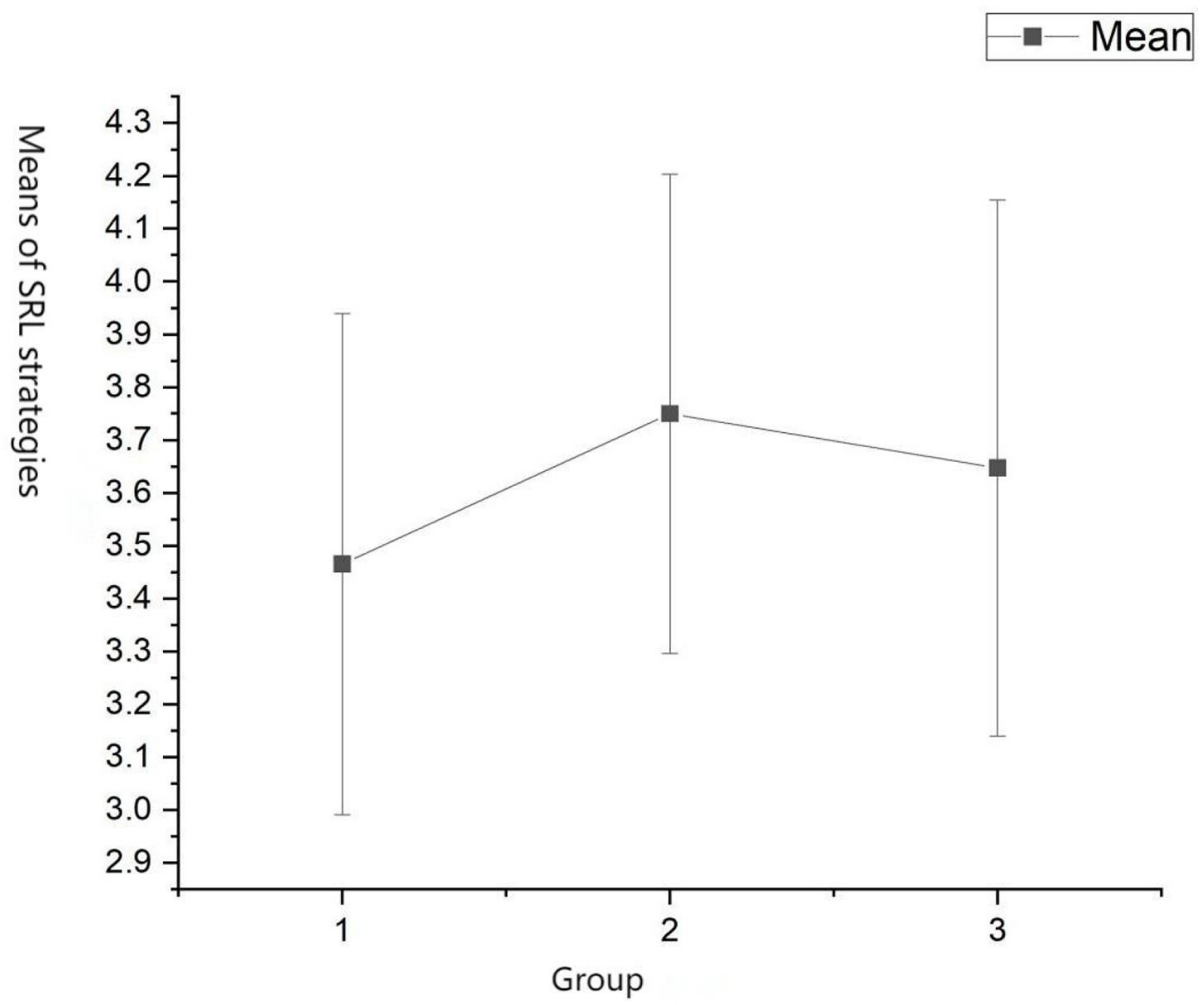
Means plot of SRL strategies.

A one-way between-groups ANOVA was conducted to explore the impact of technology tools on SRL strategies. Since the assumptions required to conduct ANOVA were met and homogeneity of variances was not violated (*p* = 0.36), the three groups (control group, Icourse group, Icourse+Pigai group) were compared (Figure 8). There was a statistically significant difference (*p* < 0.05) in SRL strategies among the three groups, *F* = 8.59, *df* = 2, *p* < 0.01 (Table 6). Despite reaching statistical significance, the actual differences in the mean scores among the three groups were minor. The effect size, calculated using η^2^, was 0.06. *Post-hoc* comparisons using the LSD indicated that the mean score in the control group (*M* = 3.47, SD = 0.47) differed significantly from that of the Icourse group (*M* = 3.75, SD = 0.45) and that of the Icourse+Pigai group (*M* = 3.65, SD = 0.51). The Icourse+Pigai group did not differ significantly from the Icourse group (*p* = 0.15).

**Table 6:** ANOVA for self-regulated learning strategies.

	**Sum of squares**	**Df**	**Mean square**	***F***	***p***
Between Groups	3.938	2	1.969	8.590	<0.001
Within Groups	63.492	277	0.229		
Total	67.430	279			

### Technology Use Factors Toward Technology-Supported SRL

**Figure 9:**
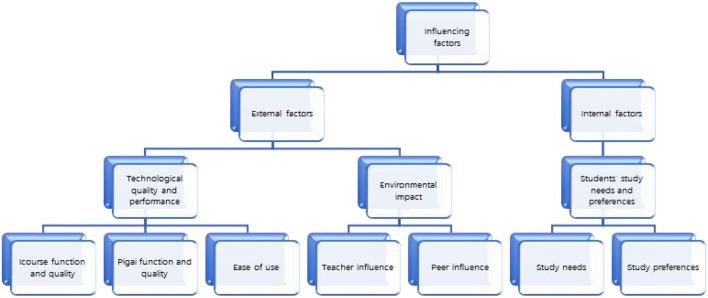
Technology use factors toward technology-supported SRL.

A list of 308 frequently occurring codes was found initially in the student transcripts and then reorganized into 46 categories of third-tier code families. Many of the themes identified in the initial coding concerned the qualities of Icourse and Pigai and the advantages and disadvantages of using the tools in academic writing. Likewise, other factors related to student needs and preferences also emerged, such as increased essay practice and unwillingness toward online peer review. As the codes were grouped and sorted, 15 categories of second-tier code families were identified, such as Icourse function, Icourse quality, Pigai function, Pigai quality, study needs, teacher influence, and peer influence. The 15 categories were then grouped as 7 broader themes and then categorized as 3 main themes and ultimately classified as two main categories as internal and external factors (Figure 9). For example, internal factors referred to study needs and study preferences, and external factors included teacher influence and peer influence.

Table 7 presents 22 categories from the 46 third-tier code families that distinctly show the differences and similarities among participants' perceptions between the two experimental groups. The five participants in the Icourse group expressed a stronger desire for an increased amount of writing practice (Icourse group: 11 citations, Icourse+Pigai: 5 citations). Compared to no complaints of Icourse drawbacks in the Icourse+Pigai group, students in the Icourse group complained about its drawbacks, such as lack of essay practice (5 citations) and inability to produce calligraphy (3 citations). Compared to the Icourse group, the most distinct feature in the Icourse+Pigai group was that students referred to self-regulated learning more frequently (Icourse group: 6 citations, Icourse+Pigai group: 10 citations). As seldom mentioned in the Icourse group, the Icourse+Pigai group referred more to the Pigai benefit of high efficiency of AWE (5 citations) and reduced teacher essay evaluation pressure (5 citations).

**Table 7:** Some technology use factors between Icourse group and Icourse+Pigai group.

	**(A) Icourse group**	**(B) Icourse+Pigai group**
1. Slack and need for supervision	8	4
2. Improve vocabulary and grammar	3	4
3. Collocation and advanced expression	6	3
4. Critical thinking and logic	0	5
5. Self-regulated learning	6	10
6. Increase the amount of essay practice	11	5
7. Academic discussions	6	9
8. Lack of essay practice	5	0
9. Unable to practice calligraphy	3	0
10. Easy access to English resources	6	2
11. Strengthen communication with teachers	3	2
12. Improve lexical complexity	2	1
13. Improve learning motivation	4	4
14. Promote knowledge gains	3	7
15. Unable to increase lexical complexity	0	3
16. AWE not intelligent enough	6	8
17. Focus on error correction	1	6
18. Reduce teacher essay evaluation pressure	1	5
19. High accuracy rates of AWE	5	4
20. AWE meticulously	7	6
21. High efficieny of AWE	1	5
22. Eases of use	3	6

## Discussion

### Writing Peformance

The Icourse+Pigai group significantly outperformed the control group in writing performance, while the Icourse group showed no significant statistical difference from the control group. The writing performance results indicate that Icourse+Pigai-mediated SRL is more conducive to enhanced writing performance than is Icourse-mediated SRL. This may be because Icourse-supported SRL fails to satisfy students' study needs for more opportunities for writing practice. As revealed by the interview results, students in the Icourse group expressed a stronger desire for frequent writing practice available through technological support.

*If there is an online system, it can be better than the current one because we are a little weaker in English writing, and then the system can give feedback and give some suggestions*. (Interviewee 1)

*I also feel that I need to practice my composition, I do feel that I don't have much practice now*. (Interviewee 2)

This research finding is consistent with Rüth et al. (2021), who found that testing and quizzes were more effective for learning than was repeated exposure to learning materials. Writing practice provides relevant cognitive load, that is, knowledge construction processes that unavoidably lead to learning (Sweller et al., 1998; Nückles et al., 2020). Pigai, which supports online writing submission and provides AWE services, enables students to learn through self-regulated writing practice. This might partially explain the higher writing scores in the Icourse+Pigai group. However, the participants perceived Icourse, the learning management system, as essential in their SRL, since Pigai does not allow exposure to learning materials, online discussion, and MOOC learning. The participants felt that Icourse and Pigai are irreplaceable because the two technological tools play their own roles in SRL.

*I think it is better to use two of them. Because Icourse supports online discussion, and then you can preview the lessons. Pigai, on the other hand, allows you to submit your essays and give feedback about your writing timely. I don't think the two conflicts with each other*. (Interviewee 9)

The participants' psychological study preferences also might have played a role in their SRL technological use.

*Since it is a writing course, I tend to have Icourse and Pigai together. I am not used to relying on only one software to study the subject. I think the two have one focus for me, so I think both of them are necessary*. (Interviewee 10)

### Lexical Complexity

No statistically significant differences in lexical complexity were found among the three groups in the pre-test. From the post-test lexical complexity results, the technology-tools-supported SRL did not significantly affect students' lexical diversity and sophistication compared with the control group. The result is consistent with the participants' interview results, in that they felt negative about Icourse and Pigai's ability to significantly improve their lexical complexity. They stated that Pigai focuses more on lexical accuracy than lexical complexity in error correction.

*It will tell you which word is misspelled, and if you misspell it, you can correct the word in your composition. In a sense, it also provides a learning opportunity. However, it does not significantly improve my lexical complexity because it cannot replace your words with more advanced words after all, and its AI technology has not yet developed to this level*. (Interviewee 7)

*I think it is more focused on picking mistakes than lexical complexity. It does not require advanced vocabulary, and it will only say that your balance of structure is relatively simple*. (Interviewee 6)

The negative perceptions are not consistent with Jia's (2016) finding that students perceived a higher level of satisfaction regarding improving their lexical complexity of writing with Pigai mediation and used a higher frequency of Basewords 2 and 3 (less frequent words) according to Range 32 software analysis. She also stated that lexical diversity and lexical density improved after a 12-week intervention. This is consistent with Zuo and Feng's (2015) result that Pigai's scoring criteria focus more on lexical accuracy than lexical complexity. Students tend to adjust their writing strategies according to the scoring criteria applied by Pigai to obtain high scores. Our results indicate that SRL supported by both the Icourse group and the Icourse+Pigai group affected students' writing performance in terms of lexical complexity but did not significantly improve on it in the current phase of technological development.

### SRL Strategies

Finally, ANOVA of SRL strategies revealed significant differences between the control and technology-supported groups. The results indicated that both Icourse and Icourse+Pigai positively related to the participants' use of SRL strategies. This aligns with van Alten et al. (2020) study, which found that providing students with technological SRL prompts is an effective strategy for improving SRL. They found that providing online videos in the process of flipped learning was positively related to students' learning outcomes. Likewise, Öztürk and Çakiroglu (2021) demonstrated that technology-mediated SRL positively enhanced students' writing skills in a flipped learning environment. According to Broadbent and Poon's (2015) review, enhanced SRL strategies positively influence learning outcome because, despite cognitive skills having a relatively negligible influence on improving learning outcomes, metacognition, time management, and critical thinking skills are positively related to learning outcomes.

However, the Icourse and Icourse+Pigai groups exhibited no significant difference in the use of SRL strategies, indicating that variation in technology tools did not significantly affect the participants' SRL strategy use. Students' psychological study preferences may partially explain this finding. Psychological study preference is compared to physical study preferences, such as, visual, aural or kinesthetic influences on study preferences. In this research, it refers to the possible psychological factors that affects students' choices on some educational modes over others. In previous studies, study preference primarily referred to sensory modality preferences. This denotes those students make study choices physically, through vision or auditory reactions (Hu et al., 2018). However, the study preferences in this research primarily referred to students' psychological factors. For instance, the interview results reflected those participants tended not to use the peer evaluation function in Pigai, even if they were told that it could be helpful to their writing. They expressed feelings of “distrust” and “embarrassment” regarding showing their essays to classmates.

*I think that sometimes it is challenging to evaluate others' work because of face issues. I just said that I still don't feel confident about my evaluation ability. I think this is a bit embarrassing*. (Interviewee 8)

*I don't think it's necessary because I think my classmates have poor writing and everyone is quite clueless*. (Interviewee 5)

Our results are consistent with van Alten et al. (2020), who found that some students disliked the SRL prompts even though the SRL support encouraged students to be more conscious of their learning. Likewise, Yot-Domínguez and Marcelo (2017) found that students tended not to use mobile-related technology tools in their SRL, but rather used mobile devices for social communication purposes. Students' psychological study preferences affect their study choices regarding technology prompts, which may subsequently influence their SRL strategy use.

### Technology Use Factors Toward Technology-Supported SRL

The research shed light on the possible factors to consider when improving students' technology-enhanced SRL experience. Findings indicate that students' perceptions toward technology-supported SRL on their academic learning of writing vary, to some degree, by both internal and external factors (Figure 9). The state-of-art technology innovations alone do not guarantee an effective learning process and outcome (Hao et al., 2021). Chew and Ng (2021) emphasized the importance of integrating the effects of students' personality and proficiency in their word contribution in online forums. Different personality traits, such as, introverts or extroverts, may lead to different word productions in their online discussion with the same technological tool (Chew and Ng, 2021). Similarly, Lai et al. (2018) reported that various external and internal factors influence students' perceived study engagement. They proposed a new perspective in viewing technological use as diversified, which means one technological use can generate multiple forms of technology supported learning experiences. Rather than viewing technology as a whole entity in itself, students' psycho-social factors are also essential in contributing to their technologically supported learning experience (Lai et al., 2018). Our research consistently supported their finding by recognizing the importance of integrating students' internal needs and psychological factors with external factors: technological quality and performance and environmental impact are equally important as technological advances in the design and implementation of technology-supported SRL for students.

## Conclusion

The study shows that the Icourse+Pigai group yielded a significantly positive result in writing performance as compared to the control and Icourse. This is partly because the Icourse+Pigai group enabled exposure to learning materials and supported more opportunities for writing practice and corrective feedback. Our research results regarding lexical complexity show that technology-supported SRL failed to significantly improve lexical diversity and sophistication. This is possibly because current feedback focuses more on lexical accuracy than on lexical complexity. Finally, the results of using SRL strategies indicated that the groups with technological support differed significantly from the control group. However, the variation in technological tools in this research did not significantly change SRL strategies. We found that students' psychological study preferences may play a role in students' choice of technological mediation of SRL strategies, all else being equal. According to student interview results, the students' perceived influencing factors were identified as external (technological quality and performance, environmental impact) and internal (study needs and preferences). We thereby conclude that it is feasible to apply both learning management systems and AWE platforms to support students' SRL learning to improve their writing performance. We call for more efforts to design technology tools that improve both lexical accuracy and lexical complexity. We conclude that the technological tools applied in this research are positively related to SRL strategies. However, students' psychological study preferences should be considered when designing technologically mediated SRL activities.

The limitations of this research lie in the heavy reliance on students' self-report questionnaires in data collection. Self-reports are sometimes biased, which reduces their validity. Future studies may add more instruments such as observation or eye-tracking techniques to triangulate the data. Furthermore, the limitations also include the possible influence of different lecturers on the group due to individual differences. Further studies may use one lecturer to teach the three groups to minimize the possible effects of the individual differences. Another limitation is that although all participants spent the same fixed time for SRL in lecture learning, the time of their SRL process spent on the preview and assignment after classes may be different. Further studies may find ways to record students' SRL study time or ask students to report their time use in SRL study in students' residences. Moreover, future studies may focus on other technological combinations or technology types since there is a wide range of available technological tools, such as AI and mobile technologies. Further investigation is necessary to explore the effects of psychological study preferences on technologically supported SRL strategies. Overall, a fruitful avenue for future research appears to be exploration of the effects of various technological prompts on students' SRL learning.

## Data Availability Statement

The raw data supporting the conclusions of this article will be made available by the authors, without undue reservation.

## Ethics Statement

The studies involving human participants were reviewed and approved by the University of Malaya Ethics Committee. The patients/participants provided their written informed consent to participate in this study.

## Author Contributions

YH and SZ conceived the idea and drafted the research. YH carried out the data collection and analysis. SZ and L-LN supervised the research and provided the critical feedback. All authors read and approved the final manuscript.

## Funding

The research is acknowledged by team project of Research Promotion Program (No. SIS21T01) which is partially funded by School of International Studies, Communication University of China.

## Conflict of Interest

The authors declare that the research was conducted in the absence of any commercial or financial relationships that could be construed as a potential conflict of interest.

## Publisher's Note

All claims expressed in this article are solely those of the authors and do not necessarily represent those of their affiliated organizations, or those of the publisher, the editors and the reviewers. Any product that may be evaluated in this article, or claim that may be made by its manufacturer, is not guaranteed or endorsed by the publisher.
