# Supplementary material for: How Technology Tools Impact Writing Performance, Lexical Complexity, and Perceived Self-Regulated Learning Strategies in EFL Academic Writing: A Comparative Study
Source: Front Psychol. 2021 Nov 3;12:752793. doi: 10.3389/fpsyg.2021.752793 (PMC8596490; doi:10.3389/fpsyg.2021.752793)
Supplement: Supplementary file 1 [file Data_Sheet_1.pdf]

Appendix A Self-Regulated Study Strategies

(1) Strongly disagree; (2) Disagree; (3) Neither agree nor disagree; (4) Agree; (5) Strongly agree.

1. I like what I am learning in this class.
2. The online learning platforms（or with textbook materials） relieve my writing anxiety.
3. The online learning platforms(or with textbook materials) increase my learning motivation.
4. I expect to do well in this class.
5. I am sure I can do an excellent job on the problems and tasks assigned for this class.
6. I think that what we learn is useful.
7. I think that what we learn in the class is interesting.
8. When I study for a test, I try to put together the information from class and the book.
9. When I do my homework, I try to remember what the teachers said in class.
10. I ask myself questions to make sure I know the material I am studying.
11. When I study, I put important ideas into my own words.
12. When I study for a test, I practice saying the important facts repeatedly to myself.
13. Even when study materials are dull and uninteresting, I keep working until I finish.
14. I work on practices and exercises even if I don't have to.
15. Before I begin studying with the online platforms (or with textbook materials) , I plan the things I will need to learn.
16. I use what I have learned from old homework assignments and the textbook to do new assignments.
17. I work hard to get a good grade even when I don't like a class.
18. The online platforms (or textbook materials) help me practice what I have learned.
19. I find the online platform (or textbook materials） that outlines the chapters in my study materials very useful for my study.
20. When you find questions or problems, you discuss them with your classmates through online platforms(face-to-face with classmates).
21. You ask your questions on online discussion forums or platforms (face-to-face with teachers).

Category	Emotional SRLS	Cognitive SRLS	Meta-Cognitive SRLS	Resource management SRLS
Item	1,2,3,4,5,6,7	8,9,10,11,12,13,16	13,14,15,17	18,19,20,21,22

*SRLS = self-regulated learning strategies

Appendix B Pre- and Post-Test Grading Rubrics

	Task achievement	Coherence and cohesion	Lexical resources	Grammatical range and resources
20-16	Covers the requirements of the task and presents a clear purpose; has a consistent and appropriate tone.	Logically organizes information and ideas; there is a clear progression throughout; uses appropriately all or most of the aspects of cohesion.	Uses a wide range of vocabulary fluently and flexibly to convey precise meanings, with natural control of lexical features and with no or occasional inaccuracies in word choice and collocation.	Uses a wide range of structures; all or most sentences are error-free; makes no or only occasional errors; has good control of grammar.
15-11	Addresses the requirements of the task; presents a purpose that is generally clear; there may be inconsistency in tone.	There is a clear progression throughout; uses cohesive devices effectively overall, but cohesion within and/or between sentences may be faulty or mechanical.	Uses an adequate range of vocabulary for the task, with some awareness of style and collocation; makes some errors in word choice and spelling.	Uses a mix of simple and complex sentence forms; makes some errors in grammar, but they rarely reduce communication.
10-6	Addresses the task only partially; the format may be inappropriate in places; presents a position but this may be ambiguous, repetitive, irrelevant, or not well supported.	Presents information with some organizations, but there may be a lack of overall progression; makes inadequate, inaccurate, or over-use of cohesive devices; paragraphing may be inadequate or confusing.	Uses a limited range of vocabulary; make noticeable errors in spelling or word formation; errors may cause strain for the reader.	Uses a limited range of structures; Rarely uses complex sentences and subordinate clauses, which are less accurate; makes frequent grammatical errors.
5-1	Barely responds to the task; may attempt to present one or two ideas, but there is no development.	Does not organize ideas logically; uses a very limited range of cohesive devices.	Uses a very limited range of vocabulary; has limited control of word formation and spelling; errors severely distort the message.	Attempts sentence forms, but grammar errors predominate and distort the meaning.

Appendix C The Interview Questions

1. Please briefly introduce yourself and your academic English learning programs.

2. Do you use Icourse or Pigai or both of them to support your EFL academic writing SRL? How often do you usually use them per week?

3. Do you reckon Icourse and Pigai helpful to support your EFL writing learning? Why or why not?

4. Do you think Icourse and Pigai helpful for your lexical complexity gains in your writing? Why or why not?

5. Do you think Icourse and Pigai helpful for improving your ability of word use in your writing? Why or why not?

6. Which mode do you feel better to support your self-regulated learning? Icourse, Pigai used seperately or combined together? Why?

7. What functions do you think Icourse and Pigai help you most with your English writing? Why?

8. What functions of Icourse and Pigai are most frequently used by you?

9. What are the major problems that you commonly encounter with the technology-supported SRL during the learning of academic writing?

10. What factors will impact your choices on the learning platforms of English academic writing? Why?

11. Please give some suggestions on how to make better use of the lcourse and Pigai.com to improve your English learning.
